# Whole genome analysis of multidrug-resistant *Escherichia coli* isolate collected from drinking water in Armenia revealed the plasmid-borne *mcr-1.1-*mediated colistin resistance

**DOI:** 10.1128/spectrum.00751-24

**Published:** 2024-08-21

**Authors:** Anna Karpenko, Andrey Shelenkov, Igor Manzeniuk, Nina Kulikova, Arman Gevorgyan, Yulia Mikhaylova, Vasiliy Akimkin

**Affiliations:** 1 Department of Molecular Diagnostics and Epidemiology, Central Research Institute of Epidemiology, Moscow, Russia; 2 Administrative and Management Department – Directorate, Central Research Institute of Epidemiology, Moscow, Russia; 3 Republican Veterinary and Phytosanitary Laboratory Services Center, Yerevan, Armenia; 4 Central Research Institute of Epidemiology, Moscow, Russia; Ross University School of Veterinary Medicine, Basseterre, Saint Kitts and Nevis

**Keywords:** *Escherichia coli*, colistin, One Health concept, WGS, multidrug resistance, *mcr-1.1*, conjugative plasmid structure

## Abstract

The rate of polymyxin-resistant *Enterobacteriaceae*, as well as human and animal infections caused by them, is increasing worldwide, posing a high epidemiological threat since colistin represents a last-resort antibiotic to treat complicated infections. The study of environmental niches, in particular, aquatic ecosystems in terms of genome analysis of inhabiting antimicrobial-resistant (AMR) microorganisms as reservoirs of acquired resistance determinants (AMR genes), represents a specific concern from a One Health approach. Here, we present a phenotypic AMR analysis and molecular characterization of *Escherichia coli* isolate found in municipal drinking water after an accident in the water supply system of a residential building in Armenia in 2021. CrieF1144 *E. coli* isolate was resistant to ampicillin, ampicillin/sulbactam, cefuroxime, ciprofloxacin, levofloxacin, trimethoprim/sulfamethoxazole, colistin, and tigecycline, whereas whole genome sequencing (WGS) revealed *blaTEM-1B*, *tet(A*), and a combination of *dfrA14* with *sul1* resistance determinants, which corresponds well with phenotypic resistance above. Moreover, the multidrug-resistant isolate studied harbored *mcr-1.1* gene on a conjugative 251 Kb IncHI2 plasmid, whose structure was determined using hybrid short- and long-reads assembly. CrieF1141_p1 plasmid carried all antimicrobial resistance genes revealed in the isolate and did not harbor any virulence determinants, so it could contribute to the spread of AMR genes in the bacterial population. Two copies of ISApl1 transposase-encoding element, which is likely to mediate *mcr-1.1* gene mobilization, were revealed surrounding this gene in a plasmid.

**IMPORTANCE:**

Evolutionary patterns of *Escherichia coli* show that they usually develop into highly pathogenic forms by acquiring fitness advantages such as antimicrobial resistance (AMR) and various virulence factors through horizontal gene transfer mediated by mobile elements. This has led to high prevalence of multidrug-resistant (MDR) strains, which highlights the relevancy of enhanced surveillance to monitor and prevent transmission of the MDR bacteria to human and animal populations. However, the limited number of reports regarding the whole genome sequencing (WGS) investigation of MDR *E. coli* strains isolated from drinking water and harboring *mcr* genes hampers the adoption of a comprehensive approach to address the relationship between environmental *E. coli* populations and human and veterinary infections. Our results highlight the relevance of analyzing the environment, especially water, as a part of the surveillance programs to understand the origins and dissemination of antimicrobial resistance within the One Health concept.

## INTRODUCTION

The One Health approach highlights the importance of antimicrobial resistance (AMR) as a major issue in public health, livestock, and agriculture. The increasing rate of AMR, especially in clinically significant bacterial species, has become a serious and growing global public health problem in this century, leading to limiting possible treatment options. Currently, a huge number of studies involving clinically relevant pathogens are conducted. However, effective control of the emergence and spread of AMR determinants requires a comprehensive analysis of human and animal health and environmental conditions (1). In fact, there is growing evidence of the environment’s role in the dissemination of AMR genes and strains, especially in anthropogenically impacted ecosystems. For example, human or animal waste can be discharged into water, contaminating it (2). Thus, the environment, and the agroecosystem particularly, represents an important source of AMR (3). In this sense, the aquatic environments can contribute to the spread of AMR bacteria and promote the increasing risk of transferring resistant bacteria, in particular, the representatives of *Enterobacteriaceae* family, to humans and animals.

*Enterobacteriaceae* members include important ubiquitous Gram-negative bacterial pathogens like *Escherichia coli*, *Klebsiella pneumoniae*, and many others (4), whose ability to acquire mobile genetic elements carrying AMR and virulence determinants through horizontal transfer by plasmids has become a public healthcare problem worldwide. In the recent years, a dramatic decrease in the rates of successful treatment of infections associated with multidrug-resistant (MDR) Gram-negative bacteria has occurred, especially in the cases of β-lactams and carbapenems (3), which led to further limitation of treatment options for infections caused by these bacteria. The dissemination of extended-spectrum β-lactamases (ESBL) and plasmid-mediated carbapenemases has essentially left no drug to treat such infections, except for the older and less patient-safe last-resort antibiotic like colistin (4, 5).

Colistin, also known as polymyxin E, was widely used in veterinary medicine previously, which ultimately led to the emergence of colistin-resistant isolates. Colistin resistance was earlier associated with point mutations in chromosomal genes, whereas in 2015, the first plasmid-mediated colistin-resistant gene *mcr-1* encoding a phosphoethanolamine transferase was revealed (6). Since then, 10 different *mcr* gene variants have been reported, with *mcr-1* being globally spread (7, 8). In the recent years, there was a significant number of reports regarding colistin-resistant *E. coli* strains harboring *mcr-1* gene, which were isolated from farm animals and poultry in Europe and Asia (9–12). Moreover, the number of studies reporting environmental contamination by colistin and colistin-resistant strains is growing very quickly (13, 14).

In particular, *E. coli* isolates carrying *mcr* gene variants were reported in many cases from humans, animals, food, and the environment worldwide (5). The presence of *mcr-1* and *mcr-3* genes in animals, agricultural soils, and irrigation water was revealed in Algeria (3), whereas the study from Bangladesh reported widespread dissemination of colistin-resistant *E. coli* strains carrying *mcr-1* in food, hand rinse, and surface water (4). Other reports concerning non-potable water contamination included the detection of *mcr-3*- and *mcr-5*-carrying *E. coli* in three rivers of the Western Cape of South Africa (15), a colistin-resistant *mcr-1*-harboring *E. coli* isolate from touristic coastal water in Brazil (16), and a recent report regarding two isolates carrying *mcr-1* and *mcr-3* from different rivers in Thailand (17).

In summary, colistin resistance has become an important epidemiological problem worldwide. However, only a limited number of reports concerning *mcr*-harboring *Enterobacteriaceae* in drinking water have been published.

Here, we supplement the existing data with a detailed genomic analysis of colistin-resistant *mcr-1.1*-carrying *E. coli* isolate collected from drinking water, and provide insight into resistance mechanisms and spreading pathways. We believe that our research will contribute to better management of the risks associated with further contamination of the environment by colistin-resistant bacteria.

## MATERIALS AND METHODS

### Sample collection, susceptibility testing, and DNA isolation

One *E. coli* isolate named CrieF1144 was selected for this study from the set of 11 samples collected by the Armenia Center of Hygiene and Epidemiology as a part of regular food and water monitoring activities in 2021. CrieF1144 was isolated from municipal water after an accident in the water supply system in a residential building. Worth noting, this water is usually used as drinking fresh water in Armenia, as it is supplied from glaciers and mountain rivers. Matrix-assisted laser desorption/ionization time-of-flight mass spectrometry (MALDI-TOF) with Microflex LT system and the MALDI Biotyper Compass v.4.1.80 software (Bruker Daltonics, Germany) were used for species detection, and an antimicrobial susceptibility test for CrieF1144 *E. coli* was performed using the minimum inhibitory concentration (MIC) method with a Sensititre Analyzer (TREK Diagnostics Systems, USA). The susceptibility/resistance was checked for amikacin, ampicillin, ampicillin/sulbactam, cefepime, ceftazidime, cefuroxime, ciprofloxacin, levofloxacin, trimethoprim/sulfamethoxazole, colistin, imipenem, and tigecycline. Broth microdilution method was used for colistin resistance determination. SWIN software was applied to interpret the results in accordance with EUCAST version 11.0 (http://www.eucast.org, accessed in 2021). *E. сoli* ATCC25922 and *E. coli* ATCC35218 were used for routine internal quality control tests for MIC determination.

### Real-time PCR assay for *mcr-1*

Real-time polymerase chain reaction (qPCR) was performed using the PCR-kit «AmpliSens MDR MCR-1-FL» (Central Research Institute of Epidemiology, Moscow, Russia). The qPCR assay was performed on a Rotor-Gene 6000 (Corbett Research, Mortlake, Australia), and the data were analyzed with the Rotor-Gene 6000 v1.8 software.

### Whole-genome sequencing

Genomic DNA was isolated with the DNeasy Blood and Tissue kit (Qiagen, Hilden, Germany). Quantity was evaluated by fluorimetry with Qubit 4.0 (Thermo Fisher Scientific, USA), then the Nextera DNA Sample Prep Kit (Illumina, San Diego, CA, USA) was used for paired-end library preparation and whole genome sequencing (WGS) of the isolate on Illumina NextSeq 2000 platform (Illumina, San Diego, CA, USA).

Oxford Nanopore MinION sequencing system (Oxford Nanopore Technologies, Oxford, UK) with the Rapid Barcoding Sequencing kit SQK-RBK004 (Oxford Nanopore Technologies, Oxford, UK) was used for long-read WGS. The libraries were prepared according to the manufacturer’s protocols and were sequenced on FLO-MIN106 R9.4 flow cell with a standard 72-h sequencing protocol using the MinKNOW software version 22.03 (Oxford Nanopore Technologies, Oxford, UK).

### Genome assembly, data processing, and annotation

Base calling of the raw MinION data was performed with Guppy Basecaller version 6.4.6 (Oxford Nanopore Technologies, Oxford, UK), whereas demultiplexing was performed using Guppy barcoding software version 6.4.6 (Oxford Nanopore Technologies, Oxford, UK). Hybrid short- and long-read assembly was generated using Unicycler version 0.5.0 (bold mode, SPAdes version 3.15.4 for short reads) (18). The contigs with a length lower than 1,000 bp were discarded.

The genome assembly was uploaded to NCBI Genbank under the project number PRJNA1075679, with the genome accession number JBAGRT000000000.

Resfinder 4.3.0 software was used for antimicrobial gene detection (https://cge.cbs.dtu.dk/services/ResFinder/, accessed on 20 January 2024, using default parameters) and point mutation analysis. Virulence factors were searched in VFDB (http://www.mgc.ac.cn/VFs/main.htm, accessed on 20 January 2024, using default parameters). Plasmid replicon types, predicted mobility, and other plasmid annotation features were revealed using MOB-suite (19) with default parameters (mob_typer version 3.1.2). Plasmid visualization was created using BRIG (version 0.95, https://github.com/happykhan/BRIG, accessed on 20 February 2024).

Insertion sequences were searched in the ISfinder database (https://www-is.biotoul.fr/index.php, accessed on 26 February 2024).

The cgMLST profile building and comparison with reference data, as well as plasmid comparison, were performed using BacWGSTdb server (20) (http://bacdb.cn/BacWGSTdb/index.php, accessed on 22 January 2024).

Plasmid multilocus sequence typing (MLST) analysis was performed using Plasmid PubMLST sequence definitions (21) (https://pubmlst.org/bigsdb?db=pubmlst_plasmid_seqdef, accessed on 24 January 2024).

CRISPRCasFinder (22) was used to check for the presence of CRISPR/Cas systems and spacers in the genome. Anti-CRISPR elements were searched in AcrBank (http://cefg.uestc.cn/anti-CRISPRdb, accessed on 20 January 2024). Additional data processing and output formatting were performed using the computational pipeline we developed earlier (23, 24).

## RESULTS

### Isolate typing and resistance profile

**Fig 1:**
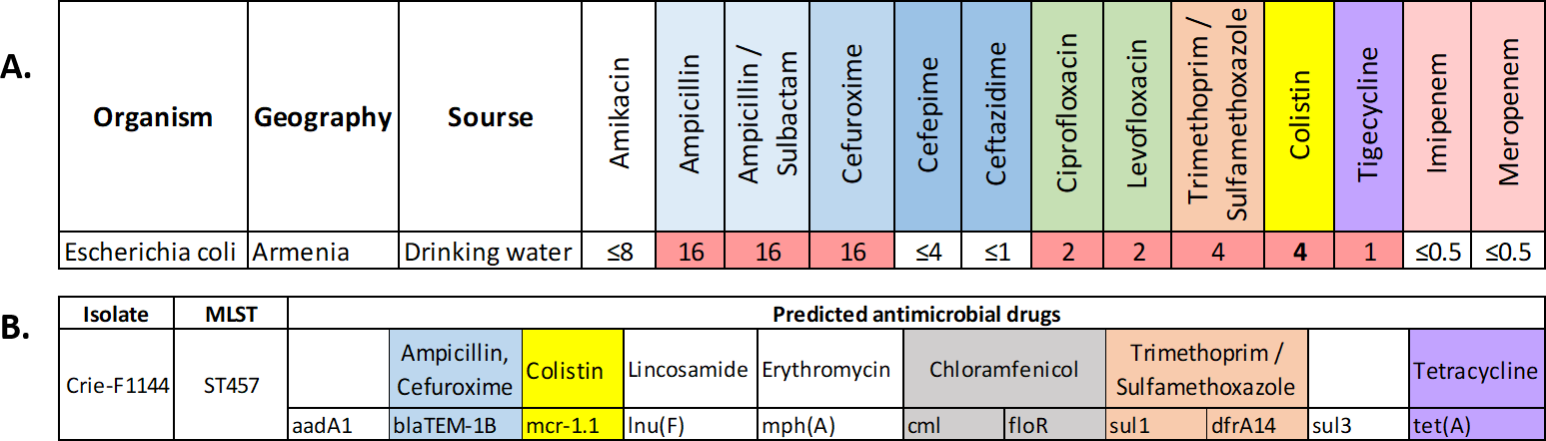
(**A**) Phenotypic antimicrobial resistance profile of MDR *E. coli* isolate from drinking water in Armenia. Red color indicates resistance; MIC, milligram per liter meanings, corresponds to EUCAST v11 standards. (**B**) Genotypic antimicrobial resistance profile of the CrieF1144 isolate from drinking water. Colors indicate different antibiotic groups: blue shows β-lactams, green is used for fluoroquinolones, yellow for polymyxins, pink for carbapenems, white for aminoglycosides, purple for tetracyclines, and orange for sulfonamides.

*In silico* MLST typing revealed that the CrieF1144 sample isolated from drinking water in Armenia belonged to ST457. According to the antimicrobial susceptibility analysis, it was susceptible to extended-spectrum cephalosporins (cefepime and ceftazidime), aminoglycosides (amikacin and gentamicin), and carbapenems (imipenem and meropenem), and resistant to the other groups of antimicrobial compounds tested (Fig. 1A). Moreover, the MIC for colistin was 4 µg/mL, which indicates strong resistance properties for the isolate studied because it is two times higher than the reference value provided by the EUCAST standard, version 11.0.

*In silico* search for antimicrobial resistance determinants provided the results that corresponded well to the phenotypic profile (Fig. 1). For example, the presence of *blaTEM-1B* gene corresponds to ampicillin resistance, *tet(A*) gene provides resistance to the group of tetracyclines, and combination of *dfrA14* with *sul1* confers resistance to trimethoprim-sulfamethoxazole. Moreover, phenotypic resistance to colistin determined for the isolate studied was attributed to the *mcr-1.1* gene revealed by WGS, the presence of which was additionally confirmed by the specific qPCR. At the same time, no mutations known to provide colistin resistance were revealed in the *pmrB* gene. Besides, *lnu(F*) and *mph(A*) were additionally found, which could be responsible for lincosamide and erythromycin resistance, respectively.

Acquired ciprofloxacin resistance genes were not revealed in CrieF1144, but the mutations in the chromosomal genes *gyrA* (S83L, D87Y), *parC* (S80I), and *parE* (S458A) shown to provide such a resistance (25) were found.

In order to obtain additional information regarding bacteria similarity, a search for similar isolates in terms of complete genome sequences was conducted. The closest isolate in terms of cgMLST profile (presented in Table S1 for CrieF1144) was UFZG01 from the UK, uploaded to Genbank in 2018. CrieF1144 and UFZG01 had only four allele mismatches and, thus, can be considered as belonging to one clone according to criteria proposed by Schürch et al. (26). Unfortunately, no data were provided regarding the source of isolation for this sample. Two other close isolates contained 37 (SWME01, Thailand) and 40 (JAAJTG01, Lebanon) allelic mismatches, respectively. Both of them were clinical samples. Other reference isolates from NCBI Genbank included more than 80 allelic mismatches and, thus, were not considered to be similar to our isolate.

### Virulence profiling

Virulence genes can be located on the chromosome or plasmids and are involved in a complex of bacteria–host interactions. The set of virulence determinants for the isolate studied was quite extensive (97 genes) and is presented in Table S2; all of them were located on the chromosome. The major virulence factors are given in Table 1. Operons of adhesion, nutrition, invasion, and effector delivery system proteins were identified among them. In addition, Type VI Secretion System (*tss*), responsible for inter-bacterial communication and survival (27), and outer membrane porin *ompA* associated with ion transport, conjugation, and host-pathogen interactions (28) were found.

**TABLE 1:** Virulence profile of colistin-resistant *E. coli* isolate CrieF1144

Isolate	MLST	Function	Localization	Virulence genes
CrieF1144	ST457	Nutrition/metabolic factors	Chromosome	*chuASTUVWXY*
				*entABCDEFS*
				*fepABCDEG iucABCD*
		Bacterial communication and survival	Chromosome	Type VI Secretion System (*tssAMLJ*)
		Effector delivery system	Chromosome	*espL1R1 × 4Y2Y4*
		Adherence	Chromosome	*fdeC*
				*fimABCDEFGHI*
				*yagVWXYZ/ecpEDCBA*
				*ykgK/ecpR*
		Invasion	Chromosome	*ibeBC kpsCDEFMSU*

### Plasmid typing, classification, and annotation

Plasmids are important subunits in dissemination of antibiotic resistance and virulence genes via horizontal gene transfer. We identified the plasmids from four incompatibility groups in the CrieF1144, the characteristics of which are shown in Table 2. IncHI2 megaplasmid had a length of about 251 Kb, whereas another plasmid, IncHI1B, had a size of almost twice less. Additionally, IncI2 plasmid was identified, and Col(KPHS6) was the smallest one. Only IncHI2 plasmid carried AMR genes, whereas no AMR or virulence determinants were revealed in other plasmids.

**TABLE 2:** Plasmids revealed in the CrieF1144 *E. coli* isolate

Plasmid name	Plasmid replicon	Size	GC content	Relaxase type	mpf type	Predicted mobility
pCrieF1144_1	IncHI2	251,464	0.47	MOBH	MPF_F	Conjugative
pCrieF1144_2	IncHI1B	97,763	0.48	-	-	Non-mobilizable
pCrieF1144_3	IncI2	59,984	0.42	MOBP	MPF_T	Conjugative
pCrieF1144_4	Col(KPHS6)	1,308	0.48	-	-	Non-mobilizable

The smallest plasmid’s nearest neighbor was pJJ1886_1 from *E. coli* JJ1886 (Genbank accession CP006785). According to the MOB-typer tool, Col(KPHS6) was predicted to be non-mobilizable, as well as IncHI1B, which had a similarity with the p266917_2_02 plasmid from *E. coli* (CP026725.1). Conjugative IncI2 was similar to a plasmid from *Shigella flexneri* (CP034061.1). The most interesting megaplasmid (pCrieF1144_1) was also conjugative, despite the significant size, and was aligned perfectly to *E. coli* plasmid 2 from UFZG01 isolate. Other similar plasmids of comparable length included pCFSAN061771_02 (CP042898.1) and p19M12 (KY689632.1), although they lacked some resistance genes revealed in pCrieF1144_1.

Hybrid assembly allowed us to determine the refined plasmid structure and obtain the data regarding AMR and virulence gene location, which represents an important step in revealing the possible mechanisms of their transfer.

Below, we will focus on the IncHI2 megaplasmid carrying AMR genes.

**Fig 2:**
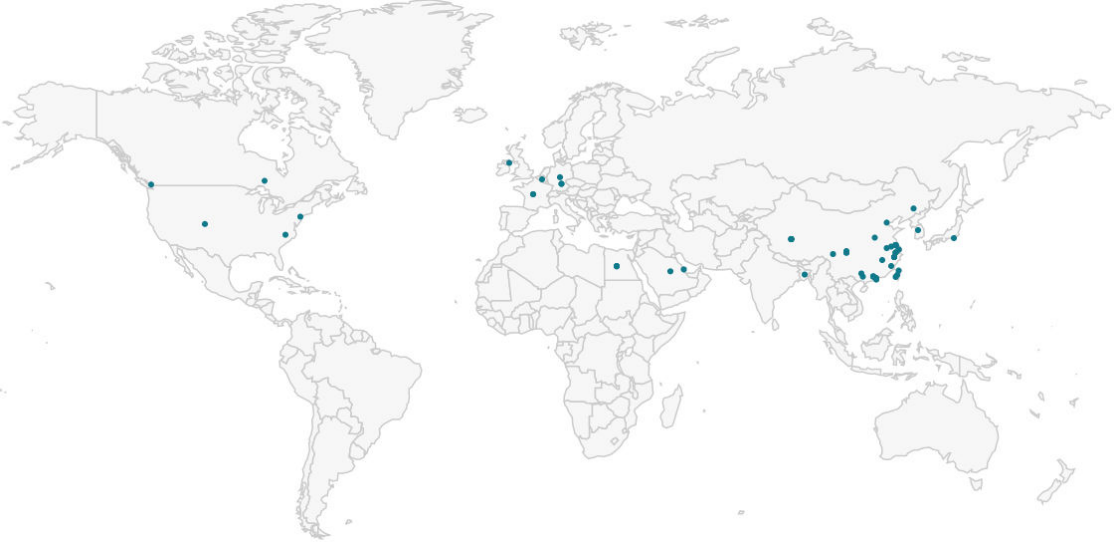
The geographic locations of reference plasmids from Genbank similar to the pCrieF1144_1 IncHI2 plasmid.

The geographic location for a large part of closely related plasmids was China (see Fig. 2). The plasmids were possessed by *E. coli* and *Salmonella enterica* isolates of human, animal, and food origin. In total, 66 plasmids carried the *mcr-1.1* gene, and one of them - *mcr-3.2* gene (p131681 [MH114596.1]). All these plasmids were selected for further comparison if they possessed the IncHI2 replicon type, and the complete list with typing results can be found in Table S3.

The double-locus sequence typing (DLST) typing of IncHI2 revealed smr0018 = 2 and smr0099 = 2 alleles for pCrieF1141_1 plasmid. It is interesting that the reference plasmids possessing the same allele variants did not carry *mcr* genes, whereas the ones with smr0018 = 3 or 4 usually carried *mcr-1.1*.

It is also interesting that IncHI2 plasmid carried an anti-CRIPR protein AcrIIA7 targeted against II-A system. CrieF1144 did not possess CRISPR-Cas system, and, in general, *E. coli* isolates possessed either I-E or I-F systems (29), whereas II-A is more common for streptococcal species (30). The map from Figure 2 was generated by BacWGSTdb server (http://bacdb.cn/BacWGSTdb/index.php, accessed on 22 January 2024).

**Fig 3:**
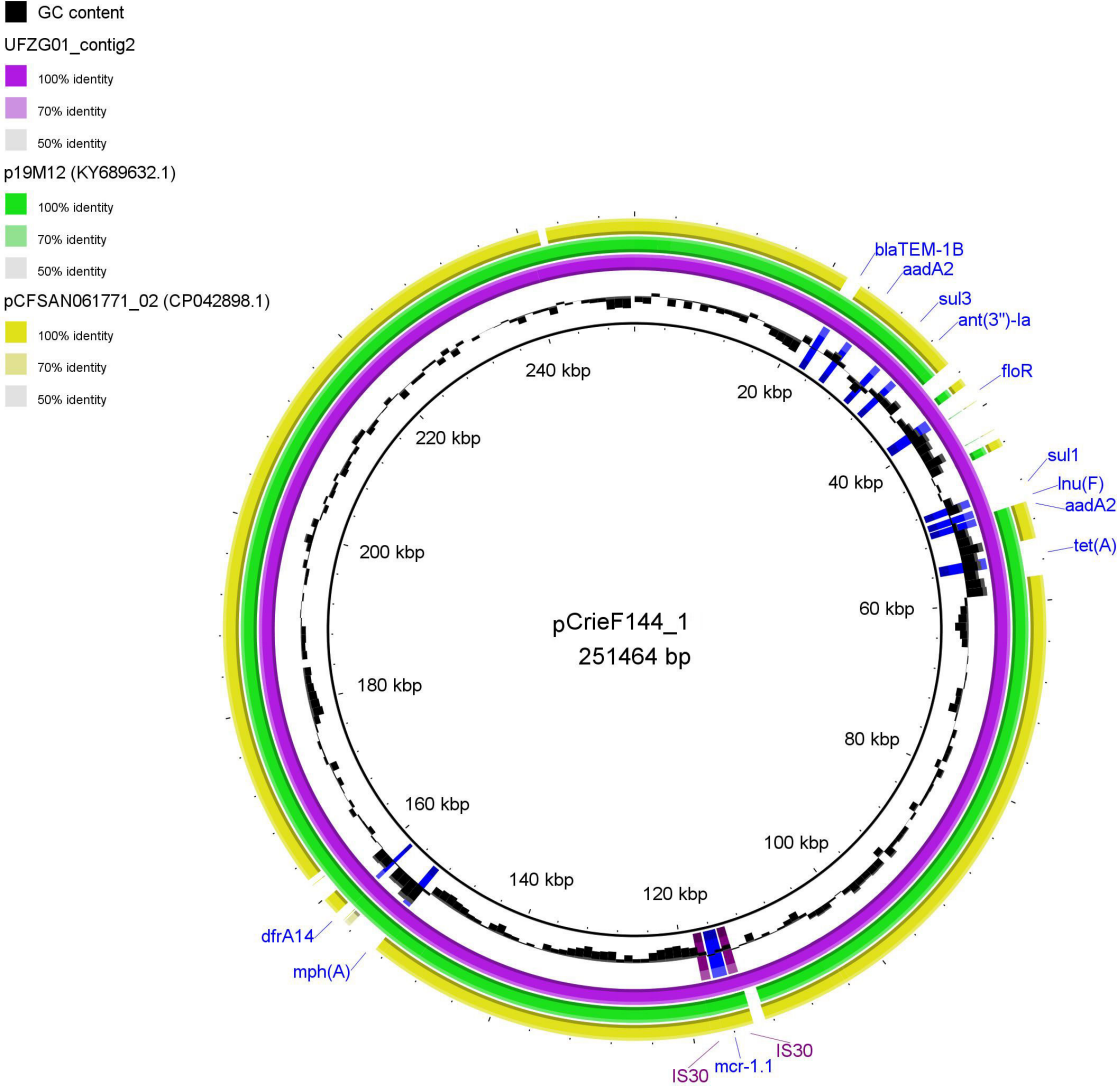
Comparison of the structures of pCrieF1144_1 plasmid and reference plasmids UFZG01_contig2 (purple), p19M12 (green), and pCFSAN061771_02 (yellow). AMR genes and insertion elements of pCrieF1144_1 are indicated.

The structures of pCrieF1141_1 and similar reference plasmids are given in Figure 3.

The plasmids pCrieF1144_1, UFZG01_contig2 (purple), p19M12 (green), and pCFSAN061771_02 (yellow) are shown. AMR genes carried by pCrieF1144_1 are indicated. UFZG01_contig2 carried exactly the same set of genes, whereas two other plasmids missed some AMR determinants. We found that *mcr-1.1* gene in pCrieF1144_1 was flanked by two transposase-encoding insertion elements ISApl1 belonging to the IS30 transposon family.

## DISCUSSION

Antibiotic-resistant bacteria pose a significant threat to public health worldwide. In particular, the emergence of plasmid-mediated resistance to colistin in MDR *Enterobacteriaceae* is dangerous due to decreased effectiveness of polymyxins representing the last-resort drugs for the treatment of infections caused by carbapenem-resistant Gram-negative bacteria. The plasmid-borne colistin resistance genes (*mcr*) represent a serious threat due to possible dissemination between different bacterial species through horizontal gene transfer. The widespread usage of colistin in livestock and the presence of *mcr-1*-positive *E. coli* strains in the food chain are considered to be the main reasons for the emergence of *mcr-1*-carrying *E. coli* in clinical cases (31, 32). The aquatic environment acts as a connection medium between different environments, thus playing a critical role in the dissemination of AMR in nature, humans, and other animals (33, 34).

On the one hand, water represents an important vehicle for the introduction and spread of drug-resistant organisms in nature; on the other hand, it is an ideal environment for the horizontal exchange of AMR genes (35, 36). Accordingly, several studies have reported the detection of resistant bacteria in rivers (15, 17), lakes (37), and sea water (16). In addition, direct transmission of clinically significant bacteria with high levels of antibiotic resistance to humans through the river water has been reported (38). Moreover, farm wastewater and damaged sewer pipes also represent possible ways of transferring AMR bacteria to groundwaters (33). Poisoned surface and groundwater can lead to contamination of purification filters and drinking water in general. Thus, it is essential to detect such bacteria in drinking water representing an important source of AMR transmission to humans (39). For a better understanding of the role of such environments and dissemination routes of *mcr* genes, we performed detection and characterization of the colistin-resistant *E. coli* isolate, named CrieF1144, carrying plasmid-mediated *mcr-1.1* gene revealed in drinking water collected in Armenia.

All virulence genes in the CrieF1144 isolate were located on the chromosome, which agrees with other studies (40). The main functions of the 96 determinants found included adherence, invasion, nutrition and metabolic factors, delivery systems, survival, and bacterial and host–pathogen interactions.

CrieF1144 belonged to ST457, which represents a globally disseminated *E. coli* lineage with a broad host range revealed in all continents (41). ST457 isolates obtained from clinical patients (42, 43), wildlife, and poultry (44, 45) were also frequently reported to carry plasmid *mcr* genes.

*In silico* analysis of AMR determinants in CrieF1144 revealed a combination of *dfrA14* with *sul1*, *blaTEM-1B*, and *tet(A*), besides *mcr-1*, which corresponds well with phenotypic resistance revealed in susceptibility tests. Such isolates co-harboring multiple AMR genes are of special concern because they may further limit treatment options for *E. coli* infections (46). Results similar to our findings were obtained in Japan (47), in which the *mcr-1* carried by *E. coli* isolates from wastewater treatment plants was revealed. At the same time, numerous studies reported a dissemination of *mcr-1* gene by *E. coli* among humans, animals, and food products. For instance, a PCR analysis revealed *mcr-1* in 62.5% of samples isolated from production animals in Poland, particularly, from turkeys (48). Moreover, a dual mechanism of colistin resistance, namely production of *mcr-1* and amino acid substitution in *PmrA/PmrB*, was detected in clinical isolates from Egypt (49).

Several *mcr*-positive *E. coli* isolates were obtained from rivers in Thailand (17) and Africa (15), surface water in Bangladesh (4), and irrigation water in Algeria (3). However, these reports did not include the plasmid data. Currently, the plasmids are considered as the major vehicles for horizontal gene transfer of the *mcr-1* variants (50).

IncHI2 is a common type of large *mcr-1*-carrying plasmids found worldwide (51), and such plasmids play an important role in the *mcr-1* transmission (52). To the best of our knowledge, this is the first report of an IncHI2 carrying the *mcr-1* gene revealed in *E. coli* from drinking water in Armenia. Previously, *mcr-1*-positive IncHI2 plasmid-harboring *E. coli* strains were revealed in clinical samples from Egypt (49), Vietnam (53), and China (54), and in food-producing animals from China (55), Poland (48), and other European countries (56).

We suppose that the *mcr-1.1* gene carried by IncHI2 plasmid in CrieF1144 plays a major role in colistin resistance of this isolate for two reasons. First, no known mutations providing such a resistance were revealed in *pmrA*/*pmrB*, *phoP*/*phoQ*, and *mgrB* genes. Second, we revealed two transposase-encoding IsApl1 elements flanking *mcr-1.1* in the plasmid sequence, which were shown to mediate the mobilization of this gene leading to resistance acquisition (57). The presence of such plasmid-encoded transposable elements raises concerns of fast resistance spreading within the environmental bacterial population.

Thus, the hybrid assembly of the genome studied allowed us not only to reveal the accurate structure of the chromosome and plasmids, but also to gain insights into possible colistin resistance mechanisms for the CrieF1144 isolate.

In summary, this study focuses on high-resolution genomic analysis of the *E. coli* isolate from drinking water, aiming to understand the genetic structure and pathogenesis of this environmental strain. As the prevalence and dissemination of MDR *E. coli* is a significant concern in the context of both human and animal populations, a comprehensive One Health-based approach is necessary to study this important pathogen. This approach involves the interconnections between human, animal, and environmental health, and establishes the collaboration between various scientific and medical fields to mitigate the growing threat of AMR spread and associated risks in urban environments (37). In the current study, we performed WGS-based characterization and phenotypic AMR analysis of the *E. coli* isolate obtained from drinking water, and revealed the possible mechanism of colistin resistance transfer. The results emphasize the importance of WGS-based monitoring of *mcr*-harboring *Enterobacteriaceae* in both clinical and environmental settings in agreement with the One Health concept.

## Data Availability

The genome assembly was uploaded to NCBI Genbank under the project number PRJNA1075679, with the genome accession number JBAGRT000000000.

## SUPPLEMENTAL MATERIAL

Table S1 spectrum.00751-24-s0001.csv cgMLST profile for CrieF1144 *Escherichia coli* isolate.

Table S2 spectrum.00751-24-s0002.csv List of virulence determinants found in CrieF1144.

Table S3 spectrum.00751-24-s0003.csv List of reference plasmids used in CrieF1144_p1 analysis.

ASM does not own the copyrights to Supplemental Material that may be linked to, or accessed through, an article. The authors have granted ASM a non-exclusive, world-wide license to publish the Supplemental Material files. Please contact the corresponding author directly for reuse.
